# Case Report: Bowel obstruction caused by an adult fibrosarcoma located in the pelvis

**DOI:** 10.3389/fsurg.2025.1721886

**Published:** 2025-12-19

**Authors:** Sixian Wang, Yiming Liu, Jixin Zhang, Junling Zhang, Xin Wang

**Affiliations:** 1 Department of General Surgery, Peking University First Hospital, Peking University, Beijing, China; 2 Department of Pathology, Peking University First Hospital, Peking University, Beijing, China

**Keywords:** adult fibrosarcoma, bowel obstruction, diagnosis, retroperitoneal soft tissue sarcoma, surgical oncology

## Abstract

**Background:**

Adult fibrosarcoma (FS) is a rare subtype of soft tissue sarcoma (STS). These tumors most commonly occur in the extremities and present as painless, enlarging masses; primary FS arising whin the abdominal cavity or pelvis represents an exceptionally uncommon entity, with limited documented cases.

**Case presentation:**

We report a case of primary pelvic adult FS in a 52-year-old male who presented with persistent abdominal pain and bowel obstruction. Abdominal Computed Tomography (CT) revealed a large, round, mixed-density mass occupying the pelvis, with suspected involvement of adjacent organs. Final histopathological diagnosis of adult FS was confirmed following comprehensive immunohistochemical workup.

**Conclusion:**

This case dramatically underscores the critical importance for gastrointestinal surgeons and oncologists to maintain awareness of rare STS like FS in patients presenting with unexplained abdominal symptoms or obstruction. Early recognition, facilitated by advanced imaging and meticulous pathology, and the multidisciplinary management strategy are paramount to optimizing outcomes in these diagnostically challenging and aggressive tumors.

## Introduction

Sarcomas are a rare and heterogeneous group of solid tumors of mesenchymal origin accounting for only 1% of all adult malignancies and 15% of childhood malignancies (1). Accounting for only 3.6% of soft tissues sarcomas (STS), fibrosarcoma (FS) is a kind of ultra-rare sarcomas and can be classified into adult-type and infantile-type based on age and biological behavior (2). Previously considered the most common sarcoma in adults, many cases have since been reclassified with the advances in diagnostic technology and the identification of other tumor morphologies that resemble FS (2). Currently, adult-onset fibrosarcoma is typically diagnosed through exclusion, due to its distinctive clinicopathological characteristics and treatment. Adult FS is most common in middle-aged and older adults (median age 50 years) and there is a slight male preponderance of 1.6:1 (3). To evaluate the prognosis of STS, the French Federation of Cancer Centers Sarcoma Group (FNCLCC) system is recommended considering the degree of differentiation, the mitotic count, and the amount of tumor necrosis (4). The overall survival rate of FS is <70% at 2 years and <55% at 5 years regardless of grade (2). FSs are primarily found in the extremities, trunk, head, and neck, but they can originate in the abdominal cavity, breast, kidney and corpus cavernosum (5–8). To the best of our knowledge, adult FS located in the pelvis have not been previously reported. Due to their proximity to critical structures, it presents a unique challenge to achieve microscopically negative margins (R0 resection) compared to tumors located in the extremities or retroperitoneal space. We present a case of high-grade adult FS in the pelvis of a middle-aged patient, along with our diagnostic and surgical approach.

## Case report

**Figure 1:**
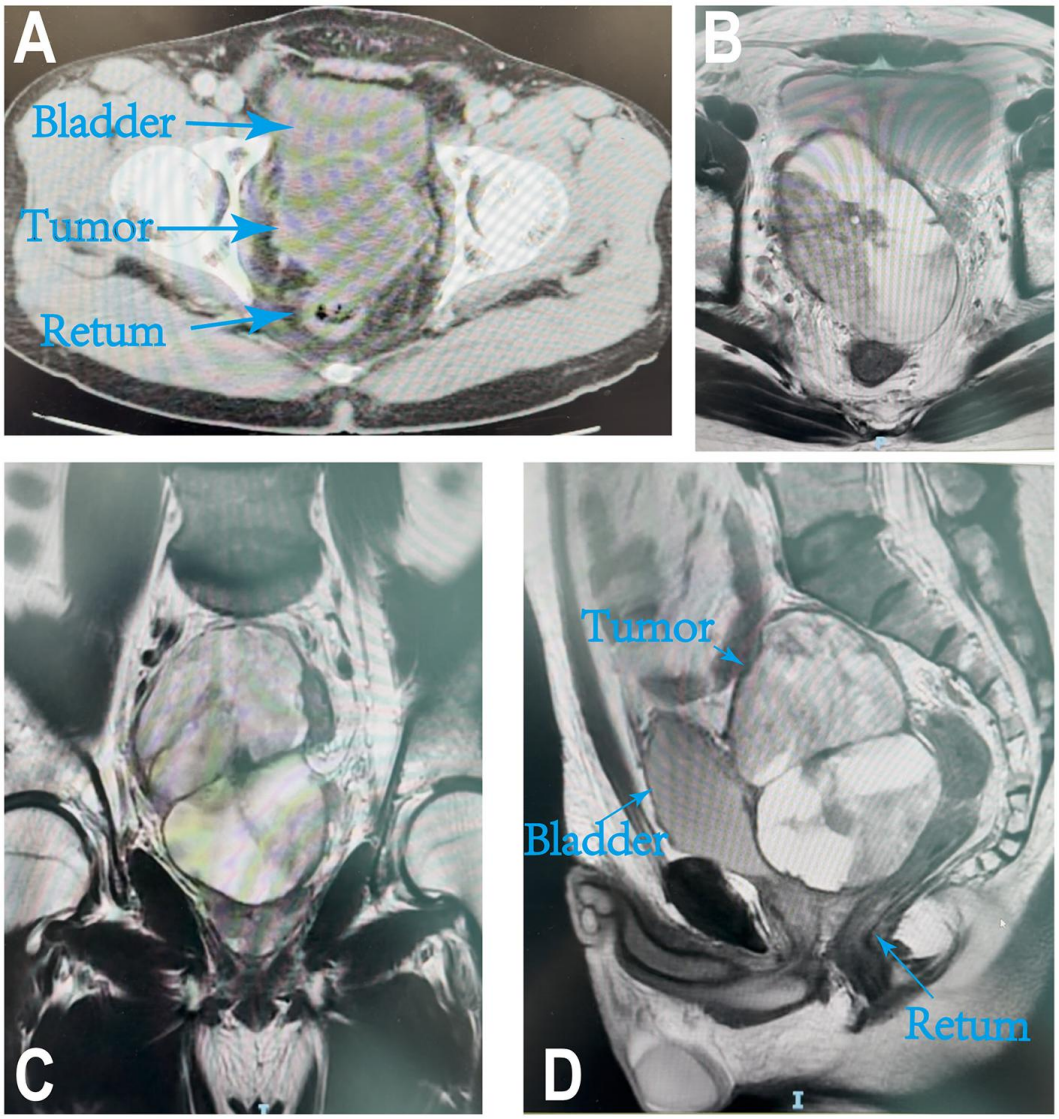
Computed tomography and magnetic resonance images performed at the first hospital. **(A)** An oval-shaped mass is visible within the right pararectal and retro vesical spaces of the bladder, measuring approximately 9.3 × 6.3 × 7.2 cm in overall dimensions. **(B–D)** The axial, coronal, and sagittal MRI images revealing a mass located in the pelvic.

**Figure 2:**
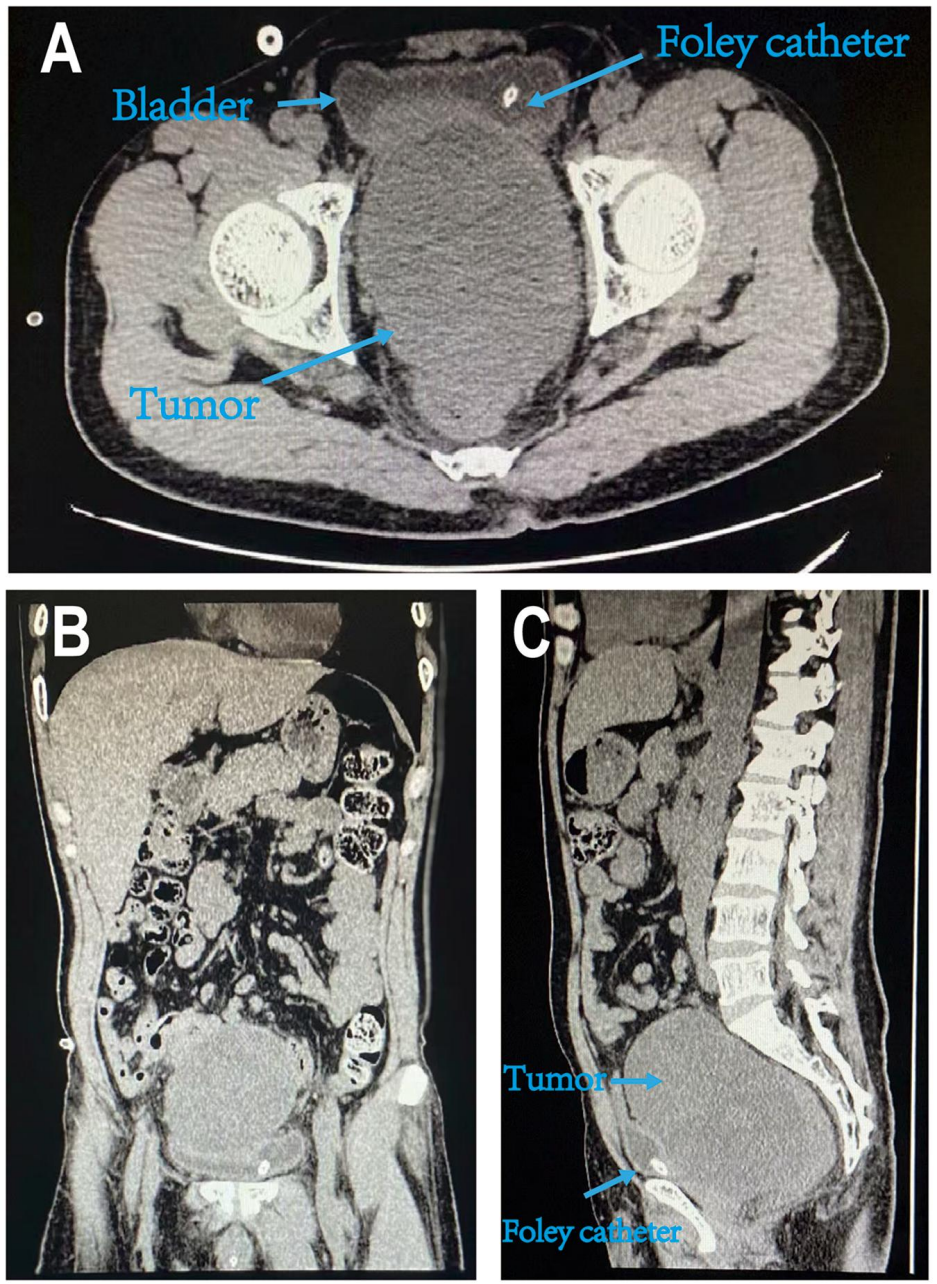
Abdominal plain CT scan image due to the sudden-onset pain and dysuria. **(A–C)** The axial, coronal, and sagittal CT scans revealing a round soft tissue mass located on the right side of the rectum and behind the bladder.

**Figure 3:**
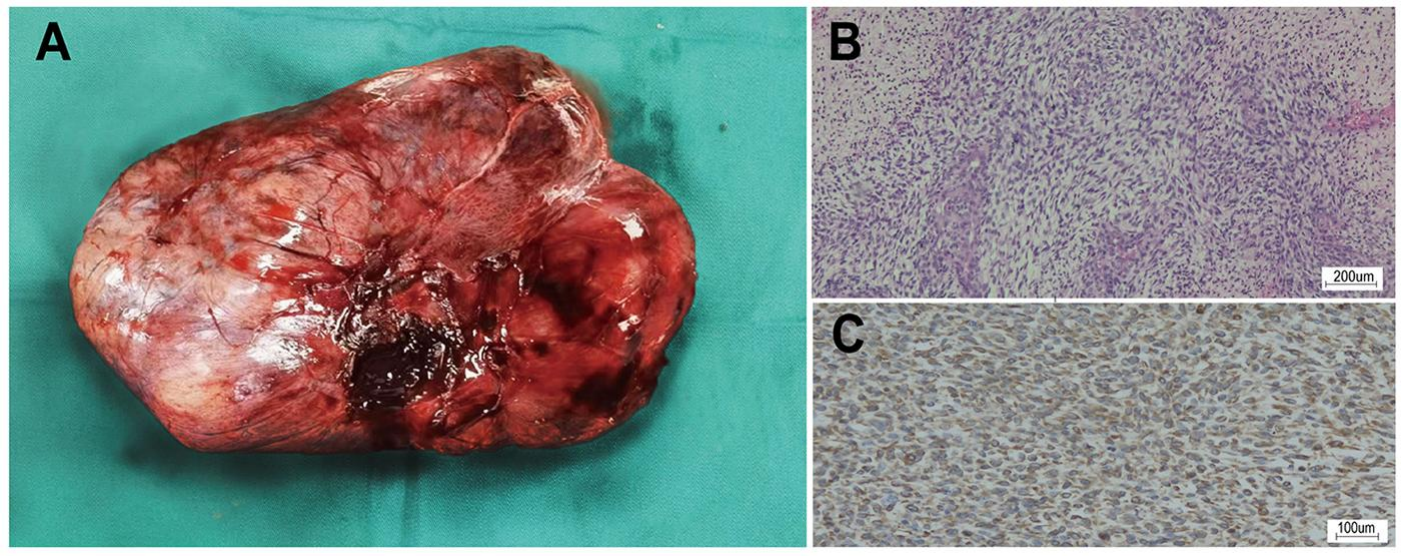
The macroscopic and microscopic images of the tumor. **(A)** An elliptically shaped tough mass. **(B)** Medium magnification view (200×) showing spindle cells arranged in interwoven bundles with moderate to severe cytologic atypia. **(C)** The tumor cells exhibit positive reaction to Vimentin on immunochemical stains.

**Figure 4:**
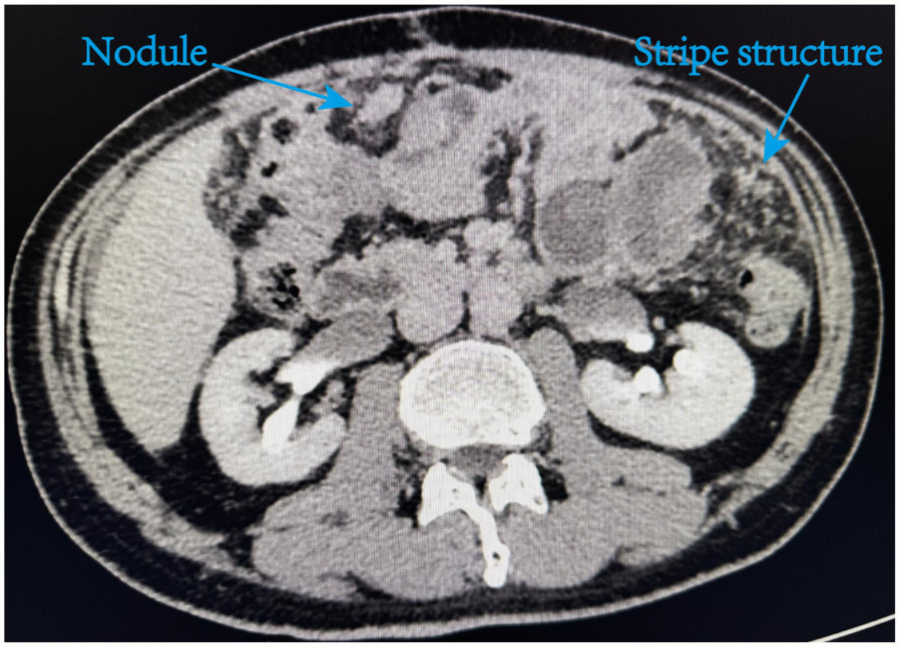
Abdominal CT image performed 3months after surgery revealed multiple metastatic lesions within the abdominal cavity. The omentum shows scattered nodules and cord-like masses.

A 53-year-old male presented to our outpatient clinic with complaints of lower abdominal pain and a pelvic mass, which had been present for approximately one month. He also reported difficulty with defecation, although he was still able to pass flatus. His stool had become thinner and less frequent, occurring approximately every two to three days, without any signs of hematochezia or melena. He did not experience other symptoms, such as fever, vomiting, hematuria, or jaundice. The patient had no history of previous surgeries and denied any comorbidities or regular use of medication. Employed as a security guard at a local hospital, he reported no exposure to radiation or chemicals. His family history was negative for cancer. He does not smoke but has consumed three ounces of alcohol daily for approximately thirty years. His initial consultation took place at another hospital, where he underwent abdominal ultrasound, contrast-enhanced computed tomography (CT), and magnetic resonance imaging (MRI) examinations. The abdominal ultrasound revealed a cystic-solid mass located posterior to the bladder, with further investigation needed to differentiate between prostate and other potential origins. The CT and MRI examination, performed at another medical center, identified a large tumor occupying the pelvis, adjacent to both the bladder and rectum (Figure 1). The radiologist initially suspected a mesenchymal origin and the hospital advised him to wait for further admission instructions. Consequently, he brought his images and sought further consultation and treatment at our outpatient clinic. For further diagnosis, we arranged a urology consultation and an experienced urologist performed an ultrasound-guided transrectal needle biopsy of the mass, which is considered to be a high-grade spindle cell tumor. We recommend a multidisciplinary consultation and admission for further treatment. One month later, during which he waited for admission, he presented to our hospital's emergency department with sudden worsening abdominal distension and pain as well as a newly-onset symptom, dysuria. The emergency physician performed urinary catheterization, collected blood samples for testing, and conducted an abdominal plain CT scan. The blood count indicates a total white blood cell count of 8.09 × 10⁹/L, with neutrophils accounting for 81.8%. The hemoglobin levels are 116 g/L and the C-reactive protein is 56 mg/L. As for his nutritional status, the albumin level is 40.2 g/L, while the prealbumin level has decreased slightly to 195 mg/L. No significant abnormalities were observed in coagulation function or other electrolyte results. Compared with the CT scan taken two months ago, the pelvic mass has increased in size and is now compressing the bladder and rectum, whilst the upper ureter shows dilatation and hydronephrosis. A mixed-density lesion measuring approximately 10.2 × 12.3 × 11.6 cm was located on the right side of the rectum and behind the bladder (Figure 2). After the emergency physician administered a glycerol enema, there was no significant improvement in the patient's abdominal pain and bloating. Consequently, the patient was instructed to fast and receive total parenteral nutrition. Following five days of conservative treatment with no notable improvement, the patient was admitted to the inpatient department for further management. Upon the patient's admission to the ward, a multidisciplinary consultation was convened, involving the departments of oncology, radiology, urology, and anesthesiology. Based on the patient's MRI, CT, and histopathological reports, we initially diagnosed the mass as a soft tissue tumor. In accordance with the National Comprehensive Cancer Network (NCCN) guidelines, we decided to proceed with surgical resection as the first step because there were no pulmonary metastases on the chest x-ray examination and the patient was suffering from bowel obstruction as well as dysuria caused by the mass. The other examinations such as electrocardiogram, ultrasonic cardiogram, and carotid ultrasound were all normal. Owing to the substantial size of the mass and the patient's mild anemia prior to surgery, the anesthetist assessed the procedure as high risk (American Society of Anesthesiologists, ASA grade 3) and required the patient to be transferred to the surgical intensive care unit postoperatively. The patient underwent a total pelvic exenteration, which included the removal of the rectum, bladder, prostate, and bilateral seminal vesicles, as they were involved by the tumor. The entire procedure lasted approximately six and a half hours, during which the patient lost about 2,500 mL of blood. To correct the anemia, six units of packed red blood cells were transfused intraoperatively. Macroscopically, the tumor tissue was an oval shape and measured appropriately 15 × 10 × 9 cm (Figure 3A). There was no evidence of malignancy in the lymph nodes; however, the bladder was involved by the tumor. Histologically, the short, spindle-shaped tumor cells, which exhibited moderate to severe atypia, were arranged in bundles and braided patterns (Figure 3B). The tumor demonstrated high mitotic activity (28/10 high-power fields) and contained less than 50% tissue necrosis. Additionally, the immunohistochemical study showed positive staining for Vimentin, S-100, and p53. No staining was detected for CD34, CD117, DOG-1, smooth muscle actin, or desmin (Figure 3C). Based on the combined cytomorphologic and immunohistochemical results, the tumor was diagnosed as an adult FS. The nuclear fission pattern (28/10 HFP), intratumoral necrosis (<50%), and conventional fibrosarcoma morphology were assigned scores of 3, 1, and 2 points, respectively, resulting in a total score of 6 points. Thus, the FNCLCC grade was classified as III, indicating a poor prognosis. The patient was transferred to the surgical intensive care unit immediately after surgery for further management. A postoperative blood count on the same day revealed a hemoglobin level of 88 g/L. The patient was transferred back to the general ward on the second postoperative day and began oral intake on the seventh day without experiencing any discomfort. He was discharged on day 10 without any postoperative complications. Despite complete surgical resection with tumor-free surgical margins, he experienced a tumor relapse with peritoneal metastasis three months later. The abdominal CT scan reveals diffuse increased density in the greater omentum, with multiple cord-like structures and visible nodules (Figure 4), suggesting metastasis. Finally, the patient declined chemotherapy or radiotherapy and died six months later.

## Discussion

Adult FS usually occurs in the deep soft tissues of the extremities, trunk, head, and neck. In reported cases, the tumor has also been found to involve the transverse colon (5), breast (6), kidney (7) and corpus cavernosum (8), among others. Most lesions typically present as asymptomatic masses in the extremities or trunk; however, when located in the viscera, they may cause additional symptoms such as abdominal pain. In our case, the patient exhibited symptoms of bowel obstruction and dysuria, which were caused by the large tumor compressing surrounding organs—a presentation not previously reported.

Accurate diagnosis is crucial for determining the primary treatment strategy for patients with STS. Generally, intra-abdominal spindle cell proliferations include mesenchymal tumors, particularly gastrointestinal stromal tumors, non-mesenchymal tumors such as malignant melanoma and sarcomatoid carcinoma, and metastatic sarcomas from other sites, like the pelvic and extra-abdominal organs (9). A thorough history and physical examination are essential for the initial evaluation. Radiological imaging is important for assessing the tumor's extent, guiding biopsy, staging the disease, determining the diagnosis, and planning therapy (10). Both CT and MRI provide detailed information about the anatomic extent of the tumor and its relationships with vital structures. MRI is the preferred imaging modality for extremity sarcomas, accurately evaluating anatomic compartment and individual muscle involvement (11). It can determine the tumor's growth, size, margin, signal density, homogeneity, and distribution of contrast accumulation (12). The lesions usually demonstrated a lobulated long T1 weighted image (T1WI) and mixed T2 weighted image (T2WI) signal mass, with band-like areas of long T1WI and short T2WI signal indicating tumor fibrous septa (13). Contrast-enhanced scans showing heterogeneous peripheral enhancement and spoke-wheel-like enhancement are highly suggestive of adult FS. Morphologically, the relatively monomorphic spindle cells showing no more than moderate nuclear pleomorphism and a fascicular, herringbone architecture with variable collagen production are critical for diagnosis (2). Immunohistochemistry for FS shows a lack of expression of markers other than vimentin or very minimal smooth muscle actin (3). For optimal therapy, this diagnosis should be made with utmost care, only when all other mesenchymal and non-mesenchymal mimics have been excluded through a combination of detailed morphological study and ancillary testing.

The NCCN Guidelines for Soft Tissue Sarcoma have divided the management of STS into four parts based on the location of lesions or common pathological types (1). Here we highlight the management of retroperitoneal soft tissue sarcoma (RPS) and the current roles of surgery, radiation, and chemotherapy.

Treatment options should be decided by a multidisciplinary team with extensive experience in the treatment of patients with RPS, based on the patient's age, performance status, comorbidities, location, and histologic subtype of the tumor (1). Surgical resection with appropriately negative margins is the cornerstone of treatment in the absence of metastatic disease (14). A complete resection with R0 resection is recommended to increase long-term disease-free survival (15). If surgical margins are positive on final pathology, re-resection to obtain negative margins should be strongly considered if R0 resection is a reasonable possibility and the patient status allows a safe intervention of this operation (1). The current data for radiotherapy are conflicting; an international randomized controlled trial didn't show benefit with preoperative radiation, but in the largest retrospective study to date, radiotherapy was associated with improved overall survival (OS) compared with surgery alone in either the neoadjuvant or adjuvant setting (16, 17). Postoperative radiotherapy (RT) is associated with higher rates of long-term treatment-related side effects. The current guidelines discourage the use of adjuvant radiation and recommend that neoadjuvant RT can be considered for selected patients with RPS who are at high risk for local recurrence (LR) (1, 16). Evidence regarding chemotherapy regimens used for RPS is mostly based on data of extremity STS that have included a small number of patients with RPS. Thus, the role of neoadjuvant and adjuvant chemotherapy in the treatment of localized RPS has not been clarified for the inconsistent findings reported from the studies. Theoretically, neoadjuvant chemotherapy can kill tumor cells as well as downstage the tumor to allow a higher rate of negative-margin resections, but multiple studies have failed to observe an improvement in OS (18, 19). The use of neoadjuvant chemotherapy or adjuvant chemotherapy is determined by the clinical physician (20). Neoadjuvant systemic therapy may be considered for selected patients, particularly in cases with a high risk of metastasis or when tumor downstaging is required to achieve resectability but it's not recommended for low-grade tumors (1). Postoperative chemotherapy is only considered in patients who are at high risk for metastatic disease based on surgical outcomes or clinical pathologic findings (1). In this case, the operation achieved R0 resection; however, recurrence was observed upon follow-up. Owing to the rarity of this condition, the post-operative outcome may be better if postoperative chemotherapy was considered. In the era of precision medicine, histology-specific research may offer improved treatment options.

As for advanced, unresectable, or metastatic diseases, chemotherapy and or RT is the preferred choice for these patients and surgery is considered for symptom control or those whose tumor become resectable after primary treatment (21). The most active chemotherapy regime in an unselected patient population is AIM (doxorubicin/ifosfamide/mesna) (22).

## Conclusion

Adult fibrosarcoma are often located in the deep soft tissues of the extremities or trunk; however, they may involve the breast, corpus cavernosum, kidney and intra-abdominal occasionally. An accurate diagnosis is necessary for the design of an adequate treatment plan. Once the lesion is suspected to be a kind of STS, a multidisciplinary management team of radiologists, pathologists, radiation and medical oncologists, and surgical oncologists is recommended. Due to the rarity and heterogeneity of STS, more randomized controlled trials are expected for guiding the management for each kind tumor.

## Data Availability

The original contributions presented in the study are included in the article/Supplementary Material, further inquiries can be directed to the corresponding author.
